# How Has COVID-19 Impacted Older Adults and Their Needs for Services?

**DOI:** 10.1093/geroni/igab046.1703

**Published:** 2021-12-17

**Authors:** Alycia Bayne, Mallory Kennedy, Emily Alvarez, Bernadette Wright, Lucy Theilheimer, Jennifer Benz

**Affiliations:** 1 NORC at the University of Chicago, Bethesda, Maryland, United States; 2 NORC at the University of Chicago, Chicago, Illinois, United States; 3 Meals on Wheels America, Arlington, Virginia, United States; 4 Meals On Wheels America, Arlington, Virginia, United States; 5 NORC at the University of Chicago, Kamuela, Hawaii, United States

## Abstract

COVID-19 has had profound effects on older adults and will have lasting impacts on their preferences and needs for services, including those offered by Meals on Wheels and other community organizations. Organizations serving older adults would benefit from insights about how to prioritize resources and services to address older adults’ needs during the pandemic and beyond. On behalf of Meals on Wheels America, NORC at the University of Chicago conducted a study to explore COVID-19’s impacts on older adults and older adults’ needs during the pandemic. We conducted two data collection activities with adults age 60 and older: a nationally representative survey with 1,535 respondents and 24 interviews. Results indicated that COVID-19 has affected older adults’ physical and mental health, social connectedness, employment, and use of services and technology. Informal networks of family members and friends are a source of assistance for 50% of older adults during the pandemic. Impacts of COVID-19 differed by income, rurality, disability status, and living situation. Findings documented the extent to which older adults had unmet needs during the pandemic, such as activities to help keep busy at home and affordable food to meet dietary needs. Survey respondents who have a lower income, are 75 and older, live alone, and who are Black were more likely to have unmet needs. Findings suggested a need to strengthen partnerships among organizations that serve older adults to address diverse needs, conduct ongoing assessments of older adults’ needs and preferences, and enhance assistance for informal support networks.

